# Radiological and Clinical Findings of Multiple Cerebellar Liponeurocytoma: A Case Report

**DOI:** 10.3389/fsurg.2021.686892

**Published:** 2021-07-07

**Authors:** Shan Wang, Xiaopei Xu, Chao Wang

**Affiliations:** Department of Radiology, The Second Affiliated Hospital, Zhejiang University School of Medicine, Hangzhou, China

**Keywords:** brain tumor, computed tomography, magnetic resonance imaging, liponeurocytoma, cerebellum

## Abstract

**Background:** Cerebellar liponeurocytoma is an extremely rare benign tumor which generally occurs in cerebellum and is almost always solitary. Multifocal cerebellar liponeurocytoma is exceedingly rare, only 8 cases has been reported so far. Herein we present the 9th case of multifocal cerebellar liponeurocytoma in a 70-year-old woman with the complete clinical course and comprehensive imaging findings.

**Case Presentation:** A 70-year-old woman presented with a history of intermittent headache for 5 years. Computed tomography (CT) and magnetic resonance imaging (MRI) of the brain have been performed and suggested a diagnosis of teratoma based on the imaging findings. After the surgical resection of the lesion, histopathological and immunohistochemical analyses revealed neuronal, glial, and lipomatous components and confirmed the diagnosis of multifocal cerebellar liponeurocytoma after surgical resection. During the 2-year follow-up period, the patient showed no signs of recurrence or metastasis.

**Conclusion:** We described the radiological characteristics and clinical course of an exceedingly rare case of multifocal cerebellar liponeurocytoma in the cerebellar vermis and temporal lobe. The clear multifocality makes this case unusual.

## Introduction

Cerebellar liponeurocytoma (CLN), which was first described in 1978 by Bechtel et al. (1), is classified as a WHO grade II tumor and is now considered as a clinicopathologic entity distinct from medulloblastoma. The major symptoms of the disease include headache, vomiting, gait disturbance, dysphonia, or cerebellar signs depending on the tumor location, and progressive visual symptoms are common in the later stages of the disease (2) due to raised intra-cranial pressure. Complete tumor resection is usually the cornerstone of management of CLN. In previous reports, CLN is almost always solitary and located in the cerebellum. This makes the presence of multifocal CLN an unusual condition, thus adds to the challenges in the diagnosis and management of the disease due to limited knowledge of the biological behaviors and clinical features. So far, only eight cases of multifocal have been reported and herein we report the 9th case of multifocal CLN in both cerebellar vermis and left temporal lobe. Details of the radiological characteristics and complete clinical course are provided and a review of the pertinent literatures is also given.

## Case Presentation

### Clinical History

A 70-year-old woman presented with unsteady walk without any obvious inducement in June 2018. She also described a history of occasionally falls, memory loss, hearing and vision loss, nausea, and vomiting for 3 months. In her previous medical history, she had intermittent headache and dizziness for 5 years. Neurological examinations at admission were unremarkable. An elevated level of neuron-specific enolase (NSE, 37.6 ng/ml) was found. Blood pressure, pulse, temperature, routine laboratory tests (complete blood count, liver function, renal function, and C-reactive protein) and other serum tumor markers were within normal ranges, including carbohydrate antigen 242 (CA242, 3.3 ng/ml), carbohydrate antigen 125 (CA125, <1 U/ml), carbohydrate antigen 153 (CA242, <0.5 U/ml), carbohydrate antigen 199 (CA199, 2.0 U/ml), alpha-fetoprotein serum (0.0 ng/ml), carcinoembryonic antigen (0.5 ng/ml), and β-human choriogonadotropin (2.6 U/L).

### Radiological Findings

**Figure 1:**
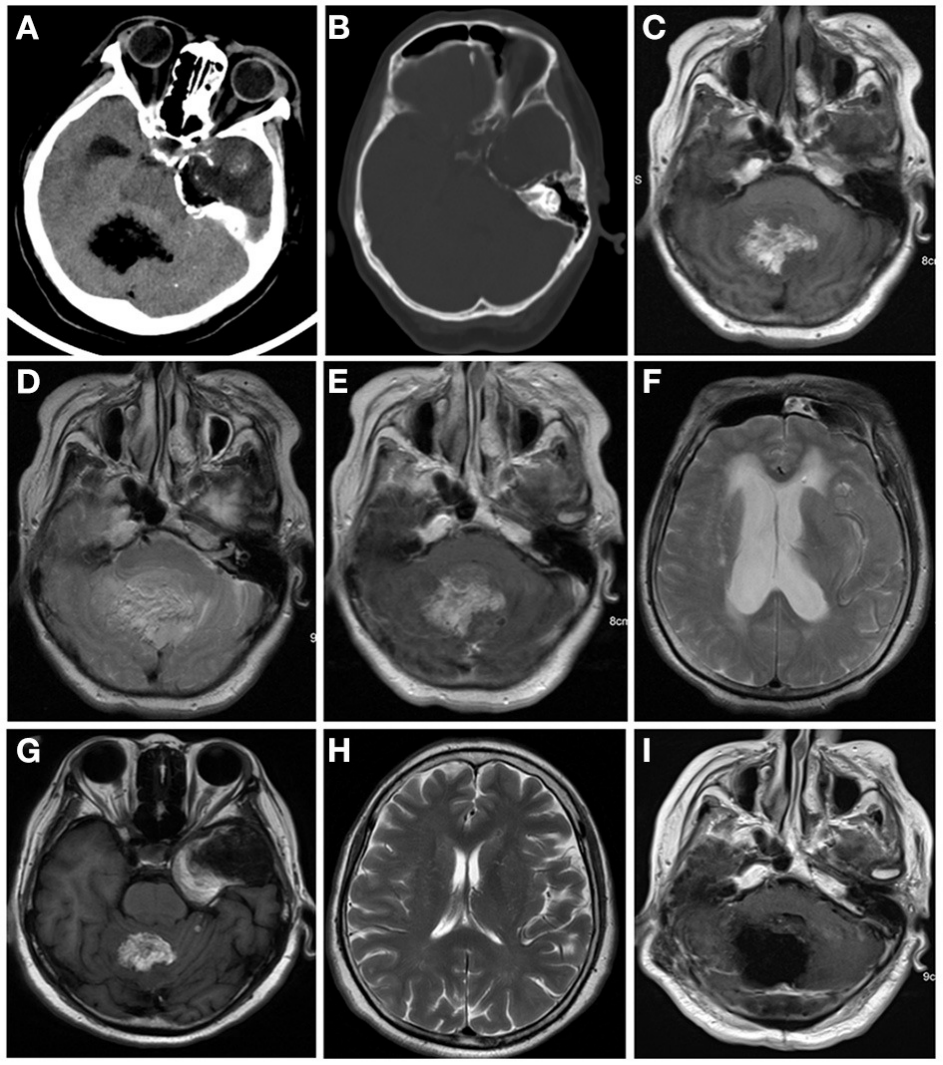
Computed tomography (CT) and contrast-enhanced magnetic resonance imaging (MRI) were performed in June 2018 **(A–E)**. CT images of brain window images demonstrated a well-demarcated mass with fat density in the left temporal lobe and cerebellar vermis with calcification **(A,B)**. MRI revealed a hyper- and isointense mixed mass on T1-weighted image **(C)** and iso- and hyperintense mixed mass on T2-weighted image **(D)**. Gadolinium-enhanced T1-weighted image showed heterogeneous enhancement **(E)**. The lesion in the cerebellum compressed the fourth ventricle **(E)** and caused supratentorial obstructive hydrocephalus **(F)**. In 2013, the cerebellum lesion was relatively small **(G)** and did not cause obstructive hydrocephalus **(H)**. Postoperative MRI indicated the cerebellar mass was near-totally removed **(I)**.

Computed tomography (CT) and contrast-enhanced magnetic resonance imaging (MRI) were performed in June 2018. CT revealed a large heterogeneous mass (5 cm ^*^ 3.4 cm) in the temporal lobe with curvilinear calcification (Figure 1A), and a small hypodense lesion (3.6 ^*^ 2.8 ^*^ 3 cm) in the cerebellar vermis with predominantly fatty density and ill-defined margin caused compression of ambient cisterns. The bone window showed the mild compression of perilesional cranial bones (Figure 1B). MRI revealed two lesions in the cerebellar vermis and left temporal lobe. The lesions were hyperintense on T1-weighted images (WI) (Figure 1C) and iso-to hyperintense on T2WI (Figure 1D), indicating the lipomatous component of the lesion. Gadolinium-enhanced T1WI revealed obvious heterogeneous enhancement, particularly in the cerebellar vermis (Figure 1E). The cerebellar lesion extended through the tentorium cerebelli and caused obstructive hydrocephalus (Figure 1F). After reviewing patient's previous medical records, a brain MRI was performed in October 2013 revealed two lesions in the cerebellar vermis and left temporal lobe. Back then, the cerebellar lesion was relatively small (6.4 ^*^ 4.3 ^*^ 4.6 cm) (Figure 1G) without any sign of obstructive hydrocephalus (Figure 1H). Judging from the radiological appearance and the benign nature of the disease, a diagnosis of teratoma was made, and vigilantly monitoring without interventions was recommended then.

### Intra-Operative Findings and Pathological Examination Results

**Figure 2:**
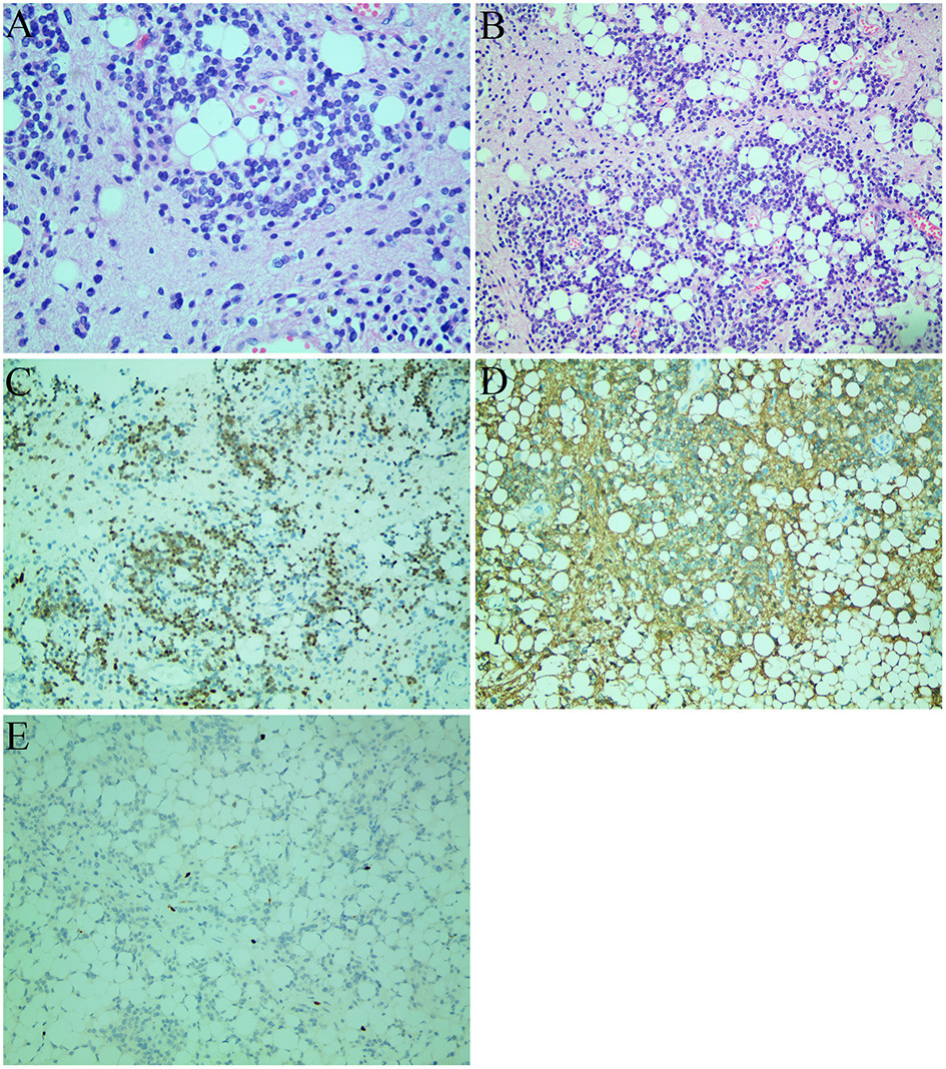
Histological images **(A,B)** and immunohistochemical staining images **(C–E)**. **(A)** The tumor consisted of uniform round cells with small, round, or oval nuclei with finely speckled chromatin and large lipid vacuoles (hematoxylin and eosin staining; original magnification: ×400). **(B)** The tumor cells were accompanied by a large number of vascular hyperplasia and papillary structure. There were scattered adipose tissue and lymphatic structures in the tumor (hematoxylin and eosin staining; original magnification: ×200). **(C)** Strong NeuN immunopositivity of neoplastic cells. **(D)** GFAP-positive component and immunonegative neurocytic fields. **(E)** The Ki-67 proliferation index was 5–10%.

Due to the unbearable headache caused by obstructive hydrocephalus, midline suboccipital craniotomy, and near-total excision of the cerebellar mass was performed, and the left temporal lobe lesions was left untreated since it is asymptomatic at time. On intraoperative examination, we observed a grayish cerebellar tumor with firm consistency, and abundant blood supply, that was not well-demarcated from the surrounding tissue. Microscopic examination showed a glioneuronal tumor with dense and diverse tumor cells, accompanied by extensive vascular hyperplasia, and papillary structures along with scattered adipose tissue and lymphatic structures in excised tumor mass (Figures 2A,B). Generally, the tumor cells had a low cell proliferation index and expressed glial and neuronal markers. Immunohistochemical staining showed the tumor cells had a positive expression for NeuN (Figure 2C), GFAP (Figure 2D), CD56, ATRX, Olig-2, and negative expression for CD45 (LCA), CD20, EMA. The Ki-67 proliferation index was low (5–10%) in tumor cells (Figure 2E). Although, the confirmed diagnosis of temporal lobe tumor was not obtained, it was considered to be liponeurocytoma because of its similar imaging features to the cerebellar mass.

### Postoperative Course

The patient was discharged 12 days after the surgery. Postoperative MRI indicated the cerebellar mass was removed near-total (Figure 1I). The temporal lobe mass was as usual. During the 2-years follow-up period, the patient had no signs of recurrence or metastasis.

## Discussion

The first case of CLN was reported by Bechtel et al. in 1978 (1). CLN is a rare neoplasm of the central nervous system with low proliferative potential. The WHO listed CLN as an independent entity in 2000 and later 2007, WHO categorized CLN as a grade II tumor (3). Although, it is named as CLN, it also occurs in supratentorial regions such as intracranial ventricles and temporal lobe (4). When it appears in the cerebellum, the clinical symptoms are usually related to intracranial hypertension and cerebellar neurological deficit (3). Patients with CLN commonly present between the third and fifth decades of life (5) and there is no gender predominance (6). Familial cases have also been reported with possible autosomal dominant inheritance (7).

CLN is composed of cells with neuronal differentiation and focal lipomatosis (8). The histopathological hallmarks are cellular tumor arranged in lobular aggregates with hyperchromatic nuclei and clear cytoplasm. In addition, CLN has other features such as brisk mitotic activity, microvascular proliferation, and foci of calcification. On immunohistochemistry analyses, CLN was positive for early and mature neuronal markers such as glial fibrillary acidic protein (GFAP), NSE, and synaptophysin. The MIB-1 labeling index tumor may elevated in some cases (9).

Our patient presented with multifocal lesions involving both temporal lobe and cerebellar vermis. Only eight cases with multifocal lesions have been previously reported. In 1997, the first case of multifocal CLNs in a 44-year-old man was reported by Horoupian et al. (10) Subsequently, seven similar cases were reported. Details of these cases are summarized in Table 1. Of the nine cases in Table 1, seven CLNs (77.8%) occurred in the cerebellum, and four CLNs (44.4%) occurred in the intracranial ventricles/cisterns. Almost all patients had headaches. Four patients (44.4%) had vomiting caused by intracranial hypertension. Among seven patients with follow-up records in Table 1, only one case (1/7, 14.2%) had recurrence. Seven out of nine cases had gone through complete surgical removal. Based on Khatri et al.'s report (17), surgical resection of the larger lesion was performed amid multiple lesions when the larger lesion was responsible for the clinical symptoms. The smaller lesions were usually left untreated and monitored regularly by CT/MRI. In 4 out of 9 cases, radiotherapy was performed yet the role of the treatment has not been well-defined.

**Table 1:** Summary of previously reported cases of multiple cerebellar liponeurocytoma.

**Authors, year (ref)**	**Age(years)/sex**	**Location**	**Symptoms**	**Surgery**	**Adjuvant therapy**	**Follow-up (months)**	**Tumor recurrence**	**Recurrence/growth after (months)**
Horoupian et al. 1997 (10)	44/M	The right lateral ventricle, the fourth ventricle	Dizziness, nausea and vomiting, episodic headaches	A transcallosal resection of the tumor arising from the septum pellucidum	3,900 cGy RT	36	No	No
Aker et al. 2005 (11)	49/F	The vermis, the fourth ventricle	Hypertension, vertigo and vomiting and progressive vision and gait disturbances	Microscopic subtotal tumor resection	36 Gy/20 WBRT	19	nm	nm
Pelz et al. 2013 (12)	54/F	The fourth ventricle, in the lateral right cerebellar hemisphere	Headaches and upper neck pain	Yes	No	24	Yes	Residual tumor 24 months after surgery after first surgery, 24 months after second surgery
Scoppetta et al. 2015 (13)	42/M	Peripheral cerebellar	Headache	No	No	nm	nm	nm
Dhar et al. 2015 (14)	49/F	Left cerebellopontine angle, cerebellar hemisphere	Headache accompanied by recurrent episodes of vomiting	Left retro-mastoid sub occipital craniotomy	No	nm	nm	nm
Konovalov et al. 2015 (15)	42/M	The head of the brain and all the vertebrae	Neck pain and bad arm feeling	Yes	RT	36	No	No
Sivaraju et al. 2017 (16)	37/M	The left side of the cerebellar vermis, the cerebellar hemispheres	Headache, vomiting, and gait disturbance	No	60 Gy WBRT	24	No	No
Khatri et al. 2018 (17)	36/F	The left cerebellar hemisphere, the right cerebellar hemisphere	Headache and difficulty in walking	Left paramedian suboccipital craniectomy	No	8	No	No
Present case	70/F	The temporal lobe, the cerebellar vermis	Headache and walking unstable	A midline suboccipital craniotomy and near total removal of the cerebellar mass	No	24	No	No

*F, female; M, male; nm, not mentioned; WBRT, whole brain radiation therapy; RT, radiation therapy*.

In our case, the relative small lesion in the cerebellum increased slowly in size and showed an aggressive feature during the 5-years follow-up period. Jenkinson et al. (18) reported a similar invasive case, emphasizing that the behavior of these tumors might be less predictable than previously thought. Long-term follow-up and further investigation of future cases is certainly necessary for a better understanding of the nature of these neoplasms.

Cerebellar hemisphere is the most common site, while fourth ventricle is the second common site for CLN. The fat composition was observed within the tumor in all cases. Calcification and hemorrhage were observed in one case, while cystic changes were found in five cases. CLN was typically isointense or hyperintense on T1WI. On T2WI and fluid-attenuated inversion recovery (FLAIR) images, the tumor was hyperintense. Heterogeneous contrast enhancement was found in nearly all cases. Edema in surrounding tissue was rare. On CT, the tumor was variably iso-to hypodense (compared to brain parenchyma) with focal areas of marked hypoattenuation corresponding to fat component (19). The key imaging feature was the fat density or signal inside the mass (20). The lesion was typically isointense/hypointense on T1WI and hyperintense on T2WI; high signal (fat signal) can be found on T1WI and T2WI/FLAIR in some areas of the mass. Sometimes edema was found around the mass on T2WI. Calcification was occasionally seen. On the contrast-enhanced scan, the mass commonly showed heterogeneous enhancement. CLN might show space-occupying effect and compress the adjacent structure. In our case, the smaller lesion compressed the fourth ventricle and caused obstructive hydrocephalus. Few tumors showed invasive nature.

Differential diagnosis of CLN includes medulloblastoma and intracranial teratoma. Medulloblastoma usually presents at younger age (<20 years old) with CT showing a slightly hyperintense lesion in the fourth ventricle. Cystic changes and calcification are commonly seen in tumors. The lesion is usually isointense or hypointense on T1WI, while hyperintense on T2WI on MRI. Intracranial teratoma are benign tumors occurring mainly in the pineal and sellar region, mostly in young men.

In conclusion, CLN is a rare intracranial tumor with unknown etiology, which can occur in the cerebellum or supratentorial parenchyma. The distinctive imaging feature of CLN is the fatty component within the lesion. An accurate diagnosis based solely on its imaging findings preoperatively is usually is difficult due to its rarity. Surgery is the primary treatment of choice for large masses causing symptoms. Long-term follow-up is needed for unresected small lesions.

## Data Availability Statement

The original contributions presented in the study are included in the article/Supplementary Material, further inquiries can be directed to the corresponding author/s.

## Ethics Statement

The studies involving human participants were reviewed and approved by the ethics committee of the Second Affiliated Hospital, Zhejiang University School of Medicine. The patients/participants provided their written informed consent to participate in this study.

## Author Contributions

CW performed the data acquisition. CW and SW performed the radiological images analysis. CW, SW, and XX performed the manuscript preparation. All authors contributed to the article and approved the submitted version.

## Conflict of Interest

The authors declare that the research was conducted in the absence of any commercial or financial relationships that could be construed as a potential conflict of interest.
