# Supplementary figures and images for: Radiological and Clinical Findings of Multiple Cerebellar Liponeurocytoma: A Case Report
Source: Front Surg. 2021 Jul 7;8:686892. doi: 10.3389/fsurg.2021.686892 (PMC8293275; doi:10.3389/fsurg.2021.686892)

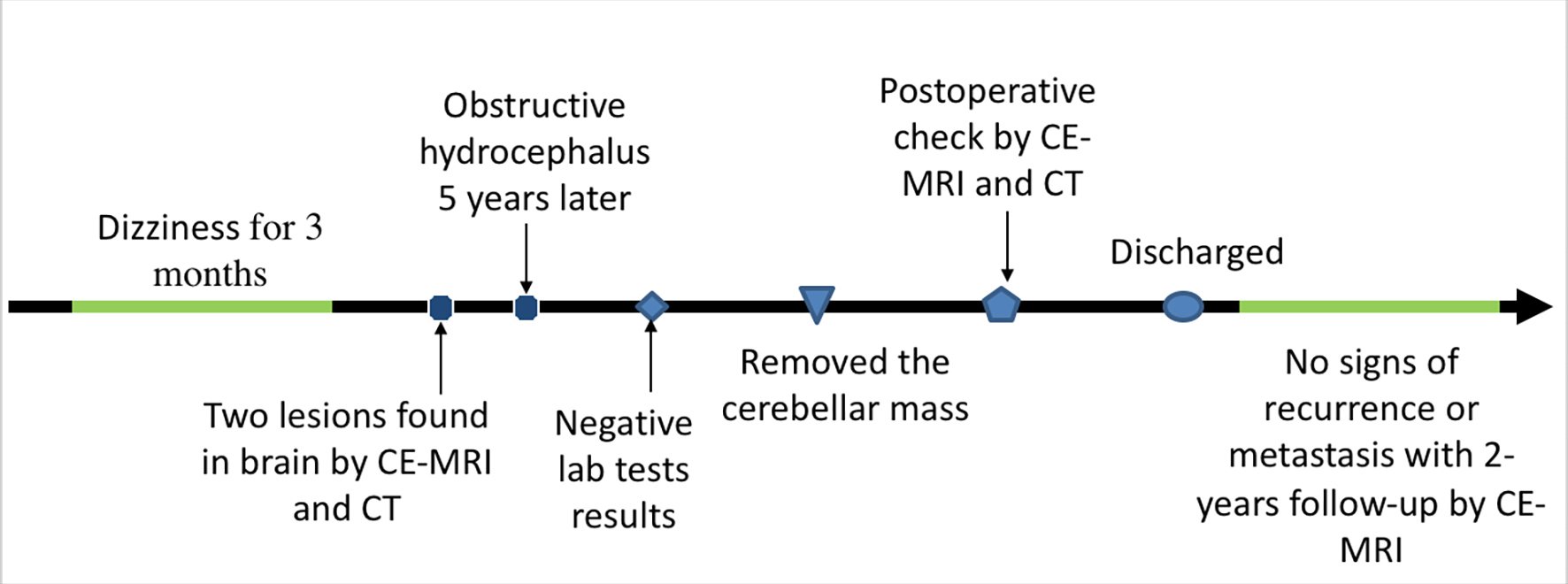

Supplement: Supplementary file 2 [file Image_1.TIF]
